# The Effectiveness of Mobile Apps for Monitoring and Management of Suicide Crisis: A Systematic Review of the Literature

**DOI:** 10.3390/jcm11195616

**Published:** 2022-09-23

**Authors:** Salvatore Sarubbi, Elena Rogante, Denise Erbuto, Mariarosaria Cifrodelli, Giuseppe Sarli, Lorenzo Polidori, David Lester, Isabella Berardelli, Maurizio Pompili

**Affiliations:** 1 Department of Human Neurosciences, Sapienza University of Rome, Viale dell’Università 30, 00185 Rome, Italy; 2 Department of Neurosciences, Mental Health and Sensory Organs, Suicide Prevention Centre, Sant’Andrea Hospital, Faculty of Medicine and Psychology, Sapienza University of Rome, Via di Grottarossa 1035, 00189 Rome, Italy; 3 Psychiatry Residency Training Program, Psychiatry Unit, Sant’Andrea Hospital, Sapienza University of Rome, Via di Grottarossa 1035, 00189 Rome, Italy; 4 Psychology Program, Stockton University, Galloway, New Jersey, NJ 08205, USA

**Keywords:** suicide prevention, mobile app, efficacy, feasibility, acceptability

## Abstract

Suicide risk is a multifaceted phenomenon, and many risk factors are involved in its complexity. In the last few decades, mental health apps have spread, providing economic and affordable strategies to prevent suicide. Therefore, the aim of this review is to identify original studies on mobile apps that target suicidal crises. The review follows PRISMA guidelines, searching through four major electronic databases (PubMed/MEDLINE, Scopus, PsycInfo and Web of Science) for relevant titles/abstracts published from January 2010 to May 2022. It includes original studies that explicitly analyze mobile apps for suicide prevention. A total of 32 studies met the inclusion criteria. Sixteen studies assessed the feasibility and acceptability of mobile apps, ten studies assessed the efficacy of mobile apps in preventing suicide, and six studies described randomized control trial protocols not yet implemented. Generally, the apps were judged by participants to be acceptable and helpful, and several improvements to enhance the functionality of apps were suggested. The efficacy of mobile apps, although limited and assessed with very heterogenous methods, was confirmed by most of the studies. Mobile apps could represent a helpful supplement to traditional prevention tactics, providing real-time monitoring of at-risk persons, personalized tools to cope with suicidal crises, and immediate access to specific support.

## 1. Introduction

Suicide is one of the most common causes of death in the general population and is the fourth leading cause of death among people aged 15–29 years old [1]. In 2019, more than 700,000 people died by suicide. Furthermore, for each suicide, there are more than 20 suicide attempts. [1]. Suicidal behavior is a complex phenomenon in which many risk factors interact with one another, producing great suffering for the patient [2]. Often, such complexity is confined in obsolete paradigms and single diagnostic labels [2]. The presence of a psychiatric diagnosis, however, is not a sufficient factor to fully explain the complexity of a suicidal crisis; in fact, suicidal individuals may not fit into diagnostic categories and may lack a full clinical picture [2,3,4]. Main risk factors include demographic factors, emotion regulation deficits, and social, relational and psychological features [5,6,7]. Prior suicidal behavior increases the risk of subsequent death by suicide 10- to 60-fold, and about 40% of suicide attempters die as a result of their second or later attempt [8]. Furthermore, people who have attempted suicide multiple times show different risk factors in comparison to single attempters [9,10].

The recent coronavirus disease (COVID-19) pandemic has sped the digital transformation of healthcare, and approximately 10,000 to 20,000 mental health apps are currently available [11]. Advantages of app-delivered interventions include constant availability, greater access, equity of mental health resources, immediate support, anonymity, tailored content, lower cost and increased service capability and efficiency [12]. Furthermore, smart-device apps overcome geographical barriers to treatment and may reduce barriers encountered in face-to-face help seeking, as well as reducing the stigma frequently associated with mental disorders [13]. App-delivered interventions have been shown to be useful in the treatment of several psychiatric disorders, such as depression, anxiety, substance abuse disorders and chronic insomnia [14].

Suicide ideation and attempts can be prevented by recognizing risk factors and protective factors [10,15]. Along with the suicide prevention strategies currently available, apps for suicide prevention have the potential to contribute to the reduction in suicide attempts and deaths through several types of interventions [16]. Screening for suicide risk, developing coping skills and emotional regulation strategies, providing emergency contact details, facilitating access to psychotherapy, encouraging people at risk to obtain support from family and friends, training others to recognize individuals at potential risk, and developing a safety plan are all possible interventions that apps can provide for suicide prevention [17,18,19,20]. However, evidence of the effectiveness of apps in reducing suicide ideation, plans and attempts remains unclear, and most of the apps currently available lack clinically validated evidence of their efficacy [16]. Moreover, previous systematic reviews [16,17,18] have analyzed only a small number of the articles about suicide prevention apps, providing a partial view of the available literature, while others [21,22,23,24] have directly assessed apps downloaded from the Android and iOS app stores to screen and examine the general features, the inclusion of educative elements and evidence-based assessment of suicide risk, and the strategies to manage suicidal thoughts incorporated in these apps. As highlighted by De La Torre and colleagues [24], the number of apps regarding suicide prevention is relatively small, and there is still little information available from literature searches, indicating that technology-based suicide prevention remains understudied. Given that mental health apps can benefit many individuals, either by improving their health monitoring or simply to verify their condition, it seems important to investigate the field and report updates.

Given the increasing number of commercially available mental health apps and the need to develop guidelines for apps marketed for suicide prevention [25], the current review aims to provide a contemporary assessment of mobile apps for the monitoring and management of suicide crisis and discusses the role of digital apps in preventing suicide in both non-clinical and clinical samples. We provide a systematic review of the apps that specifically aim to manage and monitor suicide crisis and prevent suicide in clinical and non-clinical samples, including published original studies on the feasibility, acceptability and efficacy of mobile apps.

## 2. Materials and Methods

This review was prepared in accordance with the Preferred Reporting Items for Systematic Reviews and Meta-Analyses (PRISMA) guidelines [26].

### 2.1. Search Strategy

We performed a systematic search in four major electronic databases containing medical and social science research papers (PubMed/MEDLINE, Scopus, PsycInfo and Web of Science) for relevant titles and abstracts published between January 2010 and May 2022. The following terms were combined to search the databases in titles/abstract (TA): “App” (TA) AND “Suicid*” (TA).

### 2.2. Eligibility Criteria

The review includes original articles that explicitly discussed the role of digital apps in preventing suicide in both non-clinical and clinical samples. The aims of the studies were heterogenous. Some of them assessed the feasibility and acceptability of mobile apps, others assessed the efficacy of these apps in preventing suicide, and lastly, some articles described protocols not yet implemented. When a title or abstract seemed to indicate an eligible study, the full-text article was obtained and carefully examined to assess its relevance for the review. Our exclusion criteria were: (a) studies published before 2010; (b) studies with abstracts that did not explicitly mention the role of digital apps in preventing suicide; (c) studies without suicide-specific outcomes; (d) studies that only investigated the presence of suicide risk without any intervention; (e) studies not published in peer-reviewed journals; (f) studies not published in English, and meta-analytical, systematic, or narrative reviews, and book chapters.

### 2.3. Study Selection and Data Collection

The authors independently extracted and reviewed the studies using a two-step process: (1) screening and selecting based on the article’s title and abstract, and (2) screening and selecting based on the full text. A data extraction spreadsheet was developed, adding the author(s), publication year, study design, sample characteristics (population type and sample size), name of the mobile app, specific features of the app and main results. Potential disagreements on article inclusion and data collection were resolved through discussions among the senior authors, who also independently read all articles.

### 2.4. Study Inclusion

**Figure 1:**
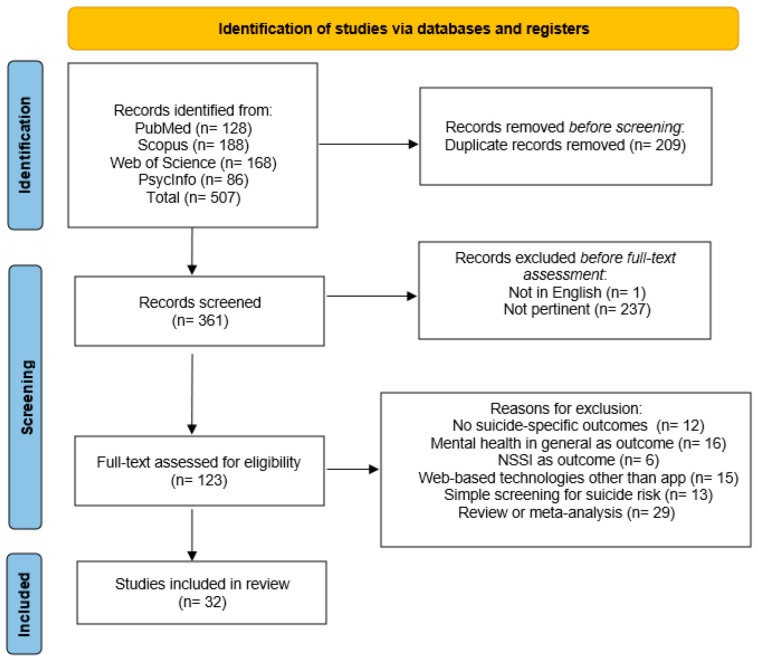
PRISMA flowchart. PRISMA, Preferred Reporting Items for Systematic Reviews and Meta-Analyses [26].

The PRISMA flowchart of the study selection process is presented in Figure 1. Electronic searches identified 570 publications. After excluding duplicates (*n* = 209), 361 studies remained. After non-English studies (*n* = 1) and non-pertinent studies (*n* = 237) were removed, 123 abstracts and titles were screened for suitability, with 91 records excluded because they were not in line with the aims and inclusion criteria of our review (See Figure 1 for detailed reasons). A total of 32 full-text articles were then assessed for eligibility via a full-text screening. Any disagreements regarding study eligibility were resolved following consensus discussions between three authors (G.S., M.C., L.P.). Overall, 32 studies were included in the present review.

### 2.5. Study Quality Assessment

The following criteria, adapted from the Study Quality Assessment Tools edited by the National Institute of Health (NIH), were used to assess the quality of the articles that evaluated the efficacy of mobile apps: (1) research question or objective clearly stated; (2) clear definition of the study population; (3) representativeness of the sample (0.5 point) and presence of clearly stated inclusion and exclusion criteria (0.5 point); (4) justification of sample size or presence of a power analysis; (5) presence of a sufficient timeframe to justify the association between usage of the mobile app and outcome; (6) presence of more than one assessment point (i.e., baseline and follow-up or pre- and post-test); (7) presence of evidence-based measures to assess the outcome (0.5 point for self-report; 1 point for clinician report, because clinician reported measures are generally considered more reliable) (i.e., 0,5 for Beck Scale for Suicidal Ideation; 1 point for Columbia Suicide Severity Rating Scale); (8) presence of assessors blinded to the status of participants; (9) less than 20% participants lost to follow-up; (10) presence of covariates adjusted statistically for their impact on the outcome. A score between 0 and 1 was attributed to each criterion, with an overall quality score ranging from 0 to 10. Co-authors first examined the selected manuscript separately; secondly, they discussed the scores together. Eventual discrepancies or disagreements were resolved by consensus with the senior researcher. After assigning a score to each study, the authors divided them into 3 categories: low-quality studies (0–3 points), moderate-quality studies (4–6 points) and good-quality studies (7–10 points). Findings from studies with a low-quality score were interpreted with caution. The mean score of all included studies was 7.1.

## 3. Results

### 3.1. Study Characteristics

The 32 publications included in the review consisted of 16 studies that examined the feasibility and acceptability of mobile apps for suicide prevention [27,28,29,30,31,32,33,34,35,36,37,38,39,40,41,42], 10 studies that analyzed the efficacy of mobile apps in managing suicide risk [43,44,45,46,47,48,49,50,51,52], of which 7 studies were randomized control trials (RCTs) [43,44,46,47,49,52], and 6 studies that described protocols of randomized control trials not yet implemented [53,54,55,56,57,58]. Publication dates ranged from 2013 to 2022. Table 1, Table 2, Table 3 and Table 4 report selected study characteristics. The majority of studies took place in Europe (*n* = 13) [31,32,33,34,35,39,41,42,43,45,49,53,54] and the USA (*n* = 9) [27,28,30,37,38,40,43,44,47].

### 3.2. Studies on Feasibility and Acceptability of Mobile Apps

Sixteen studies analyzed the feasibility and acceptability of mobile apps that aim to prevent suicidality (Table 1). The samples recruited for each study were heterogenous and consisted of psychiatric patients [30,33,34,40,41,42], adolescents with suicidal ideation or behaviors [28,31,32,39], Australian Aboriginals [29,36], students and expert clinicians [35], and transgender individuals [37]. Most of the apps presented the opportunity to create a safety plan [31,33,35], provided tools for improving coping skills [27,28,30,34,40], consisted of ecological momentary assessments (EMAs) [38,41], or allowed daily monitoring of the state of mind through a mood diary [33,37,41].

For example, the app BlueIce [32,39] provides a personalized toolbox of strategies based on theoretical approaches including Dialectical Behavioral Therapy (DBT), Cognitive–Behavioral Therapy (CBT), Mindfulness-Based Cognitive Therapy (MBCT) and behavioral activation. The Crisis Care app [28] has two versions. One version is designed for adolescents and provides coping skills. The other version is designed for parents and includes listening strategies and coaching skills. Both versions allow the user to obtain immediate help in case of need.

Regarding the feasibility and acceptability of these apps, researchers conducted different assessments: focus groups [29,31,35,37], clinical field testing [27], qualitative analysis [33,34,37,39,41], estimates of interactions with the app [28,30,33,34,36,38,41], semi-structured interviews [32,35,36,37,38,39,42] and self-report satisfaction questionnaires [28,30,38,40,41].

Although the methods used to assess feasibility and acceptability were different and the tools provided varied, all the apps were considered functional and helpful by the users, who also had the opportunity to suggest improvements and upgrades. Many studies also raised critical issues relative to the implementation of new technologies in suicide prevention. Buus et al., [31] for example, highlighted both the strengths and the limitations of the app MyPlan. The users praised the increased sense of personal control and the enhanced coping strategies, but they also highlighted the difficulties in learning how to effectively create a safety plan, suggesting the introduction of additional support for users. Data on the app BlueIce [32] brought out six key themes, and the users considered the app to be accessible, easy to use and convenient. In particular, the mood diary and mood lifter sections were considered helpful in tracking mood and in offering strategies to manage self-harm thoughts. Considering the app iBobbly [36], the authors outlined positive engagement results, concluding that the app is culturally safe and has therapeutic value. Qualitative analyses highlighted self-reported improvements in psychological well-being, mental health literacy and reductions in shame.

### 3.3. Studies on the Efficacy of Mobile Apps

All 10 studies recruited a sample with a risk of suicide or a history of suicidal ideation or behavior, except Tighe et al. [46], which was focused on Australian indigenous people. Randomized control trials (RCTs) had a control group [43,44,46,47,49,51,52], while three studies [45,48,50] were cross-sectional, considering pre- and post-test results (Table 2 and Table 3).

In terms of app content, each study presented different characteristics. The most frequent app functions were opportunities to create a safety plan [45,47,48,49,50], to develop coping skills [44,45,48,51] and to learn emotion regulation strategies [46,47,52]. For example, the Virtual Hope Box app [44] is a digital version of the hope box that clinicians suggest using when experiencing significant distress or suicidal ideation. It represents a container of items that reminds the user of positive life experiences and elicits coping resources or reasons for living. The BRITE app [47] provides access to distress tolerance strategies, emotion regulation skills and a personalized safety plan. The app sends daily messages to rate the level of emotional distress and, consequently, suggests appropriate skills and strategies to overcome difficulties. If a high level of distress is detected, the app presents the safety plan and clinical contact options. The BeyondNow app [48] allows the user to create, edit, access and share their personalized safety plan with warning signs, reasons for living, ways to limit access to lethal means, coping strategies, and personal and professional contacts. The app also includes an emergency button to quickly access an emergency service phone number and videos that explain how to complete safety plans and further information. The Therapeutic Evaluative Conditioning (TEC) [43] is a modified evaluative conditioning procedure that has an engaging, game-like design. It can be accessed by any device with an Internet connection and is meant to be played many times outside of the laboratory. It takes 1 to 2 min to complete a single instance, and it becomes more challenging as the trials progress. Points are awarded for faster and more accurate performance. The version used in the study targeted self-related words (e.g., me, myself, I, mine) and self-injurious thoughts and behavior-related stimuli.

Suicide-specific outcomes, albeit extremely heterogenous, were considered in each study. Several studies assessed suicidal ideation [43,44,45,46,47,48,52]. Franklin et al. [43], Kennard et al. [47], Melvin et al. [48] and Rodante et al. [51] focused on suicide behaviors; Jeong et al. [50] analyzed attitudes toward suicide according to the Theory of Planned Behavior, and O’Toole et al. [49] studied suicide risk in general. The measures used to conduct the assessment were particularly heterogenous. Some studies [45,47,48] used the Columbia Suicide Severity Rating Scale [59] (C-SSRS); two studies [43,51] used the Self-Injurious Thoughts and Behaviors Interview [60], while other measures used [49] were the Suicide-Status Form II-R [61] (SSF), the Beck Scale for Suicidal Ideation [62] (BSS), [44,45], the Depressive Symptom Inventory-Suicidality Subscale [63] (DSI-SS) [46] and the Suicidal Ideation Attributes Scale [64] (SIDAS) [52].

In terms of efficacy, several studies reported a statistically significant positive effect of the mobile app intervention on one or more suicide outcomes. Specifically, Bush et al. [44] reported that the average sum scores on the Beck Scale for Suicidal Ideation (BSS) [62] decreased over time in the total study population, with a statistically significant decrease relative to baseline observed at six weeks post-randomization. Franklin’s [43] analyses of three studies reported that Therapeutic Evaluative Conditioning (TEC) produced moderate reductions for all self-injurious thoughts and behaviors except for suicidal ideation. Reductions were reported for self-cutting episodes (32–40%), suicide plans (21–59%) and suicidal behaviors (33–77%). TEC effects were not maintained at the 1-month post-treatment follow-up. The results of the pilot study conducted by Jeong et al. [50] on three suicidal adolescents showed a decrease in attitude toward suicide attempts and a continuous decreasing trend of their suicide intention score, which persisted at the 7-day follow-up. Kennard et al. [47] reported no significant differences in the rates of suicide attempts after hospital discharge, although the results were in the hypothesized direction. A history of a suicide attempt moderated treatment outcome, with a stronger, albeit nonsignificant, effect of ASAP plus treatment as usual among patients with a history of suicide attempt.

The regression analyses conducted by Melvin et al. [48] showed a significant reduction in the severity and intensity of suicide ideation from baseline to post-intervention assessment. In the study by O’Toole et al. [49], a significant main effect of time on Suicide Status Form-II-R (SSF-II-R) [61] was found across the entire intervention period, where the self-reported suicide risk decreased, corresponding to a large effect size. Pauwels et al. [45] found a small and nonsignificant decrease in suicidal ideation according to the pre- and post-test results using the Beck Scale for Suicidal Ideation (BSS) [62]. The randomized control trial by Rodante et al. [51] estimated a decrease in the proportion of individuals who presented suicidal ideation for both the intervention and control groups. Tighe et al. [46] conducted a pilot RCT and reported that, although pre- and post-intervention changes were significant in the intervention group, the interaction of intervention group by time (pre-intervention vs. post-intervention) was not significant. Finally, the two-arm randomized control trial by Torok et al. [52] highlighted a significantly greater reduction in Suicidal Ideation Attributes Scale scores (SIDAS) [64] from T0 to T1 for the intervention group compared to the control condition, while there was no further significant change from T1 to T2 for the intervention condition. Overall, studies on the efficacy of mobile apps have shown mixed findings: some of them [43,44,48,49,50,51,52] highlighted a significant decrease in suicidal outcomes in the intervention sample, while others [45,46,47] showed no significant results or differences between intervention and control groups (see Table 2).

### 3.4. Studies That Describe Protocols Not Yet Implemented

Five papers [53,54,55,56,57,58] presented protocols that aimed to test the efficacy of mobile apps in suicide prevention (Table 4). All the studies were designed as randomized control trials and, for the most part, will take place in Australia [55,56,57,58]. The main suicidal outcomes are suicidal ideation [53,55,56,57,58], self-harm [54] and suicide behaviors [55]. Andreasson et al. [53] planned to recruit 273 patients with suicide risk in order to test the app MyPlan, which asks users to consent to the creation of digital safety plans, and compare them with 273 patients with suicide risk who will complete the safety plan on paper. Greenhalgh et al. [54] planned to implement the app BlueIce, which consists of a mood diary and a toolbox of strategies, in a sample of adolescents with a history of self-harm. The app LifeBuoy, developed by Han et al. [55], is based on DBT sessions and includes a mood tracker and a help button to contact emergency services. The authors planned to implement the app in a sample of 378 young adults with suicidal ideation. An evolution of the previous app is the one developed by McGillivray and colleagues [56], who are planning to conduct a three-arm RCT in a sample of 669 young Australians with suicidal ideation. The study will present an enhanced version of LifeBuoy that monitors and improves engagement with the app in order to increase its use and therapeutic benefits. Shand et al. [57] planned to test an app for enhancing emotion regulation and identification of values based on the principles of Acceptance and Commitment Therapy (ACT) and Mindfulness-Based Cognitive Therapy (MBCT). The sample will consist of 150 individuals with suicidal thoughts but without active suicidal intent. Lastly, Shand et al. [58] described a protocol to test the app iBobbly in a sample of 200 Aboriginal Australians. iBobbly presents culturally adapted content based on ACT, MBC and DBT, articulated in three modules on emotion regulation, action planning and values identification with the goal of preventing the development of suicidal ideation.

## 4. Discussion

The main objective of this systematic review was to provide a contemporary assessment of mobile apps for the monitoring and management of suicide crisis. We included 32 papers in the review: 16 studies examined the feasibility and acceptability of mobile apps for suicide prevention, 10 studies analyzed the efficacy of mobile apps for managing suicide risk, and 6 studies described protocols for randomized control trials not yet implemented.

The results showed that 16 studies analyzed the feasibility and acceptability of mobile apps that aim to prevent suicidality. Most of the apps present the opportunity for creating a safety plan, provide tools for improving coping skills, consist of ecological momentary assessments, or allow the daily monitoring of the state of mind through a mood diary. However, the samples recruited for each study were heterogenous, and the authors used different assessment tools. The results demonstrated that, although the methods used to assess feasibility and acceptability were different and the tools provided were varied, all the apps were considered functional and helpful by the users, who also had the chance to suggest improvements and upgrades.

Regarding the efficacy of apps, all 10 studies recruited a sample with a risk of suicide or a history of suicidal ideation or behavior, except Tighe et al. [41]. Randomized control trials (RCTs) used a control group [43,44,46,47,49,51,52], while three studies [45,48,50] conducted cross-sectional studies comparing pre- and post-test data. The most frequent app functions were opportunities for creating safety plans, developing coping skills and learning emotion regulation strategies. The measures used to conduct the assessment were particularly heterogenous. In terms of efficacy, several studies described a statistically significant positive effect of the mobile app intervention on one or more suicide outcomes.

Finally, five papers [53,54,55,56,57,58] presented protocols for randomized control trials to test the efficacy of mobile apps in suicide prevention.

Even though the results were promising, the studies considered are not exempt from several theoretical and practical flaws that prevent generalization of the results. Most of the studies did not fully respect evidence-based practices. Assessments were not systematic, and the studies used almost exclusively self-report instruments, which have limited validity and reliability. The social context and the supportive role of significant others is a fundamental resource to prevent suicide, but only one study presented an app that involved family members in the assessment [28]. Moreover, the use of technologies raises important ethical issues in terms of privacy and social media influence. Generally, as noted in a study by Reen and colleagues [65], there is a lack of information on privacy settings and data protection, as well as little implementation of evidence-based strategies for suicide prevention. Another problem is the implementation of the apps in different cultures, which was treated by only four studies [29,36,46,58]. Furthermore, developers should design more interactive apps for suicide prevention and adopt a user-centered design approach. As highlighted by many users in the different studies [28,29,34,38,39,40,42], the use of mobile apps should be complementary to adequate healthcare, a strong therapeutic alliance with clinicians and individualized strategies of suicide prevention.

The strength of our work was to provide a wide overview of the apps that specifically aim to manage and monitor suicide crisis and prevent suicide in clinical and non-clinical samples. In fact, we analyzed published original studies on the feasibility, acceptability and efficacy of mobile apps, while some reviews have only assessed the main features of apps available in Android and iOS stores to test their usability and engagement [21,22,23,24,66,67,68]. Our target was exclusively suicide risk, while other papers [14,69,70] included mental health in general. Moreover, we analyzed a greater number of apps to provide a comprehensive summary of the available literature [16,17,18,71]. Finally, some articles focused on technological interventions for preventing suicide that also include web-based approaches and not only mobile apps [18,72,73,74,75,76].

## 5. Conclusions

Assessing and managing persons at risk of suicide is complex and requires collaborative partnership between the affected person and her/his support network and a multidisciplinary healthcare team. Mobile apps could offer tools for real-time monitoring of at-risk persons and provide access to support whenever it is needed. However, apps should be seen as an addition to an ongoing patient–provider relationship and never as a replacement. Future lines of research should more fully explore this field in order to increase knowledge about the potential of mobile apps to prevent suicide. Although several apps for suicide prevention have been developed or are still under study [27,28,29,30,31,32,33,34,35,36,37,38,39,40,41,42,43,44,45,46,47,48,49,50,51,52,53,54,55,56,57,58,59,77,78,79], it is essential to conduct more controlled studies on efficacy according to evidence-based procedures, reliable measures and well-established outcomes to permit generalization of results and the dissemination of valid and cost-effective prevention strategies.

### Limitations

This review needs to be interpreted in light of several limitations. The results should be treated with caution because some of the studies involved relatively small samples, and the mean quality score of the study on the efficacy of mobile apps was moderate (7.1 out of 10), indicating that some studies had several flaws. Our study had the aims of providing a comprehensive and critical view of the present literature and encouraging research in this field. Moreover, the studies were extremely heterogenous in their methods, aims, suicide-specific outcomes and interventions delivered by the apps. These features prevent a fair comparison and generalization of the results and narrow the opportunity to draw solid conclusions about the efficacy and the potential benefits of mobile apps in preventing suicide. The significance of the present study is further diminished by the fact that no meta-analysis could be carried out because of the few and very heterogeneous studies included. Finally, the choice to include in the review different types of studies, in particular, the inclusion of protocols not yet implemented, somewhat impeded the successful synthesis of results, although our decision was driven by the intention to provide an overview as comprehensive and exhaustive as possible of the literature. Given the emerging field of apps dedicated to suicide assessment and prevention, more studies may be available that are not listed in this article. In addition, centers for public health and suicide awareness may develop tools that are not necessarily reported in scientific articles or are listed under headings not used in this review. Therefore, articles included in this review may be only part of the literature.

## Figures and Tables

**Table 1:** Studies on feasibility and acceptability of mobile apps.

Study	Country	Sample	App	Intervention Proposed	Results
Bruen et al., 2020 [33]	UK	Psychiatric inpatients	Strength Within Me (SWiM)	Journaling and safety plan.	This study reports on the engagement with the SWiM app, the technical difficulties the research team faced, the importance of building key relationships and the implications of using Facebook as a source to detect suicidality.
Bush et al., 2015 [27]	USA	Clinical sample of veterans	Virtual Hope Box (VHB)	Patient-tailored coping tools.	High-risk patients and their clinicians used the VHB more regularly and found the VHB beneficial, useful, easy to set up, said they were likely to use the VHB in the future and recommend the VHB to peers.
Buus et al., 2018 [31]	Denmark	Young and adult users of MYPLAN attending Danish Suicide Prevention Clinics, relatives, and clinicians	MYPLAN	Safety plan.	The app was considered helpful for learning to recognize early signs of an impending crisis, and for coping by actively finding personalized problem-solving strategies. Learning how to effectively implement a safety plan was not perceived to be simple, and additional support should be considered for MYPLAN users.
Cliffe et al., 2022 [39]	UK	University students with history of self-harm thoughts/behaviors	BlueIce	Personalized toolbox of strategies based on theoretical approaches, including DBT, CBT, mindfulness and behavioral activation.	Responses to BlueIce were very positive with students believing BlueIce to be a helpful resource that was perceived as more accessible than alternative support. Participants believed it could provide help in moments of distress as well as helping individuals learn longer-term coping skills. Others felt that BlueIce would not be adequate for some people and would be better used alongside other face-to-face support. Overall, BlueIce was considered acceptable to the students in this study.
Dubov et al., 2021 [37]	USA	Transgender individuals	TransLife	Mood logger.	Engaging, acceptable, and potentially effective mental health intervention. Participants reported that the app was easy to use and understand, supported mental self-care, promoted self-awareness and helped them to identify triggers of negative moods.
Grist et al., 2018 [32]	UK	Individuals attending child and adolescent mental health services (CAMHS)	BlueIce	Personalized toolbox of strategies based on theoretical approaches, including DBT, CBT, mindfulness and behavioral activation.	BlueIce was accessible, easy to use and convenient. Many highlighted the mood diary and mood lifter sections as particularly helpful in offering a way to track their moods and offering new strategies to manage their thoughts to self-harm.
Kiosses et al., 2022 [40]	USA	Psychiatric inpatients with suicide risk	WellPATH	List of triggers and negative emotions associated with SI and personalized cognitive reappraisal techniques to reduce negative emotions.	Study participants and their therapists reported high satisfaction with the app and provided several feedbacks for future research and development.
McManama et al., 2016 [28]	USA	Suicidal adolescents recruited from an outpatient psychiatric department and their parents	Crisis Care	Adolescent mode: coping skills + receive immediate help. Parents mode: listening strategies, coach adolescents in coping skills, receive immediate help.	Results demonstrated acceptability and usability, suggesting the utility of technological interventions, such as Crisis Care, as an adjunct to treatment for suicidal adolescents and their parents following discharge from acute care settings.
Morgiève et al., 2020 [34]	France	Patients with recent suicide attempt or suicidal ideation	Ecological Mental Momentary Assessment (EMMA)	Self-help tool for suicidal crisis management (warning signs, coping strategies, distraction activities, social support).	Patients have different clinical and digital profiles and needs that require a highly scalable, interactive and customizable app. To become a complementary tool for suicide prevention, EMMA should be integrated into existing emergency procedures.
O’Grady et al., 2020 [35]	Ireland	Students and expert clinicians	SafePlan	Mental health support and safety planning.	The feedback received from the usability testing day was largely positive. The participants perceived the main benefits of the SafePlan app to be its overall user interface design and emphasis on user confidentiality—in particular, the acknowledgment of the app’s privacy features. Small number of potential improvements were suggested.
Porras-Segovia et al., 2022 [41]	Spain and France	Psychiatric patients at high risk for suicide	MEmind and eB2	MEmind: active EMA that explores 4 areas: passive SI, negative feelings, sleep disturbances, appetite.eB2: passive EMA that provides feedback to the patient about weekly physical activity and sleep habits. It includes a mood diary module.	High participation rates; retention rates decreased steadily over the follow-up period. Passive EMA showed higher retention rates than the active EMA. Users showed a good level of satisfaction with both applications, to a greater extent for the active EMA than for the passive EMA.
Povey et al. 2016 [29]	Australia	Aboriginal and Torres Strait Islander youth	iBobbly	Based on ACT and MBCT. Self-help tool with three self-assessment and three activity modules. Self-assessment modules ask the user whether they are experiencing intrusive thoughts, including thoughts of suicide; if so, they are directed to emergency line. Three activity modules aim to help users manage upsetting thoughts and emotions, identify ideals and set realistic goals.	Three key themes emerged: personal factors, environmental factors and app characteristics. Specific adaptations, such as local production, culturally relevant content and graphics, a purposeful journey, clear navigation, meaningful language, options to assist people with language differences, offline use and password protection may aid uptake.
Primack et al., 2021 [38]	USA	Veterans hospitalized for SI or SA	Mobile Application for the Prevention of Suicide (MAPS)	EMA to identify suicide risk in the moment and to deliver treatment strategies in real time.	Veterans reported high levels of satisfaction with MAPS, and all opted to extend their use of MAPS beyond the 2-week trial period. MAPS may be a useful adjunctive to treatment as usual for high-risk Veterans by allowing patients and their providers to better track suicide risk and deploy intervention strategies when risk is detected.
Rizvi et al., 2016 [30]	USA	Patients with Borderline Personality Disorder and history of self-harm or SB	DBT Coach	The app includes content from all 4 modules of DBT skills manual (mindfulness, distress tolerance, emotion regulation and interpersonal effectiveness skills).	Results indicate good acceptability and usability of the DBT Coach with considerable between-person variability in the frequency of app use and a median use of only 11.5 times over the course of treatment and a 3-month follow-up period. Using a hierarchical linear modeling approach, analyses indicated that DBT Coach reduced subjective distress and urges to self-harm following app use. However, use of the DBT Coach was not related to any treatment outcomes, except for reductions in NSSI.
Schiffler et al., 2022 [42]	Austria	Patients with Borderline Personality Disorder	TalentLMS	The app offers DBT-based contents and downloadable worksheets to train DBT skills.	Six overarching themes were identified through qualitative text analysis: (1) experiences with DBT skills, (2) phenomenon of self-harm, (3) feelings connected with self-harm, (4) dealing with disorder-specific symptoms, (5) prevention of self-harm, and (6) attitude toward skills apps. In general, the provision of an app with DBT content achieved a positive response among participants. Despite a small change in the perception of suicidality and NSSI, participants could imagine its benefits by integrating their use of the app as a supportive measure for personal psychotherapy sessions.
Tighe et al., 2020 [36]	Australia	Aboriginal and Torres Strait Islander youth	iBobbly	Identification of feelings and thoughts, emotion regulation, identification of values ACT.	While the regression analysis in this study did not indicate a significant effect of app use on psychological wellbeing, this was predictable considering the small sample size (*n* = 18) and typically brief app use. The results on engagement with the iBobbly app were, however, positive. iBobbly app was considered to be culturally safe and of therapeutic value. Qualitative analyses demonstrated that iBobbly app use was associated with self-reported improvements in psychological wellbeing, mental health literacy and reductions in shame.

Legend: DBT = Dialectical Behavioral Therapy; Cognitive–Behavioral Therapy; SI = suicidal ideation; EMA = Ecological Momentary Assessment; SB = suicide behavior; NSSI = non-suicidal self-harm; ACT = Acceptance and Commitment Therapy; MBCT = Mindfulness-Based Cognitive Therapy; SA = suicide attempt.

**Table 2:** Studies on the efficacy of mobile apps.

Scheme	Design	Country	Sample	Main Suicidal Outcomes	Results	Quality Score
Bush et al., 2017 [44]	RCT	USA	118 veterans with SI (58 intervention group; 60 control group)	BSS; C-SSRS	Statistically significant decrease at 6 weeks post-randomization. No statistically significant differences between the two groups at any time point.	10
Franklin et al., 2016 [43]	RCT; 3 studies	Canada, USA, Australia, Europe	Study 1: 114 (55 intervention group; 59 control group); Study 2: 131 (62 intervention group; 69 control group); Study 3: 163 (78 intervention group; 85 control group); Participants were forum users with self-injurious thoughts and behaviors.	SITBI	Study 1: fewer NSSI episodes and suicide plans in intervention group; no significant differences in SI. Study 2: no significant differences in NSSI, SI or suicide plans. Study 3: fewer NSSI episodes and suicide plans in intervention group; no significant differences in SI.	6
Jeong et al., 2020 [50]	Pilot study one group pre–post test	South Korea	3 adolescents who attempted suicide		Decrease of positive attitude toward suicide, perceived behavioral control, subjective norms and suicide intent.	6
Kennard et al., 2018 [47]	RCT	USA	66 hospitalized suicidal adolescents (34 intervention group; 32 control group)	C-SSRS	No significant differences in SA. Past SA moderated treatment outcome with a strongest but nonsignificant effect of ASAP+TAU.	8
Melvin et al., 2019 [48]	Open-label single group trial	Australia	36 patients with suicide spectrum	C-SSRS	Significant reduction in suicidal ideation. Increased suicide-related coping strategies and suicide resilience.	8
O’Toole et al., 2019 [49]	RCT	Denmark	129 patients of a suicide prevention clinic (60 intervention group; 69 control group)	SSF-II-R	The TAU+APP group experienced a smaller decrease on the SFF compared to control group.	7.5
Pauwels et al., 2017 [45]	Pre–post single group	Belgium	21 individuals with suicidal ideation	BSS	Small and nonsignificant decrease in suicidal ideation.	3
Rodante et al., 2020 [51]	Pilot cluster RCT	Argentina	21 individuals with suicidal spectrum (11 intervention group; 10 control group)	SITBI	Higher but nonsignificant reduction in suicidal behavior in the intervention group compared to control group.	7
Tighe et al., 2017 [46]	Pilot RCT	Australia	61 Australian indigenous (31 intervention group; 30 control group)	DSI-SS; PHQ-9	Significant pre- and post-intervention changes in the intervention group, but the interaction group by time was not significant.	7
Torok et al., 2022 [52]	Two-arm parallel, double-blind, RCT	Australia	455 young adults with SI (228 intervention group; 227 control group)	SIDAS; PHQ 9	Significant improvements in suicidal ideation severity, but no secondary mental health outcomes, compared to the control condition.	8.5

Legend: RCT = randomized controlled trial; BSS = Beck Scale for Suicidal Ideation; C-SSRS = Columbia Suicide Severity Rating Scale; TAU = treatment as usual; SITBI = Self-Injurious Thoughts and Behaviors Interview; SI = suicidal ideation; NSSI = non-suicidal self-harm; SA = suicide attempt; SSF-II = Suicide Status Form-II Revised; DSI-SS = Depressive Symptom Inventory-Suicidality Subscale; PHQ-9 = Patient Health Questionnaire-9; SIDAS = Suicidal Ideation Attributes Scale.

**Table 3:** Intervention proposed by the studies on the efficacy of mobile apps.

Study	App	Intervention
Bush et al., 2017 [44]	Virtual Hope Box (VHB)	VHB vs. enhanced TAU The app provides patient-tailored coping tools.
Franklin et al., 2016 [43]	Therapeutic evaluative conditioning (TEC)	TEC vs. control version of TEC The app consists of a brief, game-like treatment that could be accessed by any device with an Internet connection.
Jeong et al., 2020 [50]	Brake of My Mind	Safety Plan app
Kennard et al., 2018 [47]	BRITE app	As Safe As Possible (ASAP)+TAU vs. TAU The app comprises four modules: chain analysis and safety planning; distress tolerance and emotion regulation; increasing positive affect by planning pleasant activites; and review of the skills, safety plan and app.
Melvin et al., 2019 [48]	BeyondNow	App+TAU Safety planning
O’Toole et al., 2019 [49]	LifeApp’tite	Collaborative Assessment and Management of Suicidality (CAMS)+TAU vs. TAU The app offers psychoeducation, self-rating assessment, sleep recording, appetite and stress levels, safety plan, digital hope kit.
Pauwels et al., 2017 [45]	BackUp	The app consists of safety plan, hope box and coping strategies.
Rodante et al., 2020 [51]	CALMA	DBT+CALMA vs. DBT The app provides psychoeducation and DBT strategies to cope with suicidal crisis.
Tighe et al., 2017 [46]	iBobbly ACT	IBobbly vs. waiting list The app consists of identification of feelings and thoughts, emotion regulation strategies and identification of values ACT.
Torok et al., 2022 [52]	LifeBuoy	LifeBuoy vs. LifeBuoy-C (same display but no therapeutic contents) DBT-based intervention to improve emotion regulation and distress tolerance.

Legend: TAU = treatment as usual; DBT = Dialectical Behavioral Therapy; ACT = Acceptance and Commitment Therapy.

**Table 4:** Protocols of studies not yet implemented.

Study	Design	Country	Sample	App	Main Suicidal Outcome	Intervention
Andreasson et al., 2017 [53]	RCT	Denmark	546 suicidal patients (273 each group)	MyPlan	Suicide Ideation; BSS	MyPlan (psychotherapy+safety plan) vs. TAU (psychotherapy+safety plan on paper)
Greenhalgh et al., 2021 [54]	RCT	UK	138 adolescents with history of self-harm	BlueIce	Self-harm; RTSHIA	Usual care+BlueIce vs. Usual care
Han et al., 2020 [55]	RCT	Australia	378 young adults	LifeBuoy	SIDAS; Suicidal behavior (presence/absence)	LifeBuoy vs. placebo app with the same display but no therapeutic contents
McGillivray et al., 2022 [56]	3-arm RCT	Australia	669 young adults with SI	LifeBuoy	SIDAS	LifeBuoy vs. LifeBuoy-C (app without therapeutic contents) vs. LifeBuoy digital engagement strategy (designed to enhance use and therapeutic benefit of the app)
Shand et al., 2013 [57]	RCT	Australia	150 individuals with suicidal thoughts but without active suicidal intent (75 each group)	No name	Suicidal thoughts; DSI-SS	App vs. Waiting list
Shand et al., 2019 [58]	RCT	Australia	200 Aboriginal participants without suicide risk	IBobbly	SI; SIDAS	IBobbly vs. Waiting list

Legend: RCT = randomized controlled trial; BSS = Beck Scale for Suicidal Ideation; TAU = treatment as usual; RTSHIA = Risk Taking and Self-Harm Inventory for Adolescent; DBT = Dialectical Behavioral Therapy; SIDAS = Suicidal Ideation Attributes Scale; SI = suicidal ideation; ACT = Acceptance and Commitment Therapy; DSI-SS = Depressive Symptom Inventory-Suicidality Subscale; MBCT = Mindfulness-Based Cognitive Therapy.

## Data Availability

Not applicable.
